# Seroepidemiological study of Toxoplasma gondii infection in a population of Iranian epileptic patients

**DOI:** 10.17179/excli2016-858

**Published:** 2017-03-13

**Authors:** Jalal Babaie, Mohammad Sayyah, Kourosh Gharagozli, Ehsan Mostafavi, Majid Golkar

**Affiliations:** 1 Department of Parasitology, Pasteur Institute of Iran, Tehran, Iran; 2 Department of Physiology and Pharmacology, Pasteur Institute of Iran, Tehran, Iran; 3 Department of Neurology, Loghman Hakim Hospital, Shahid Beheshti University of Medical Sciences, Tehran, Iran; 4 Research Centre for Emerging and Reemerging infectious diseases, Pasteur Institute of Iran, Tehran, Iran; 5 Department of Epidemiology, Pasteur Institute of Iran, Tehran, Iran

**Keywords:** epilepsy, Toxoplasma gondii, IgG, ELISA, seroprevalence

## Abstract

Epilepsy is one of the most common neurologic disorders. Underlying cause of epilepsy is unknown in 60 % of the patients. *Toxoplasma gondii* is an intracellular parasite which is capable of forming tissue cysts in brain of chronically infected hosts including humans. Some epidemiological studies suggested an association between toxoplasmosis and acquisition of epilepsy. In this study we determined seroprevalence of latent *Toxoplasma* infection in a population of Iranian epileptic patients. Participants were classified in three groups as Iranian epileptic patients (IEP, n = 414), non-epileptic patients who had other neurologic disorders (NEP, n = 150), and healthy people without any neurologic disorders (HP, n = 63). The presence of anti-*Toxoplasma *IgG antibodies and IgG titer in the sera were determined by ELISA method. Anti-*T. gondii* IgG seroprevalence obtained 35.3 %, 34.7 % and 38.1 % in IEP, NEP and HP, respectively. The seroprevalence rate was not significantly different among the three groups (P = 0.88). Anti-*T. gondii* IgG titer was 55.7 ± 78, 52.4 ± 74 and 69.7 ± 92 IU/ml in IEP, NEP and HP, respectively. There was not any statistically significant difference in the antibody titer between the study groups (P = 0.32). The rate of *T. gondii* infection in epileptic patients was not higher than non-epileptic patients and healthy people in the Iranian population.

## Introduction

Epilepsy is the most common neurological disorder after stroke and Alzheimer's disease affecting almost 1 % of people worldwide (Thurman et al., 2011[30]). In spite of development of advanced diagnostic methods, the underlying cause of epilepsy is unknown in more than 60 % of the patients (Bell et al., 2014[4]). 

*T. gondii* is an obligate, intracellular, parasitic protozoan that is capable of forming cysts in excitable tissues including the brain of the host. Nearly two billion of the world population have been exposed to *T. gondii* and are seropositive (Subauste, 2012[29]; Weiss and Kim, 2007[33]). *T. gondii* cysts are microscopic and invisible with MRI or CSF analysis. Tissue cysts seem to persist for the whole life of the infected host. Reactivation of the parasite in immunocompromised people can lead to serious clinical complications including encephalitis, myocarditis, severe disseminated disease and seizures (Ferreira and Borges, 2002[15]).

Recent studies indicated that *Toxoplasma* infection predisposes individuals to neurological disorders, particularly psychosis (Fekadu et al., 2010[14]). Risk of epilepsy in people with toxoplasmosis has also been discussed by many researchers (Akyol et al., 2007[1]; Ngugi et al., 2013[23]; Palmer, 2007[24]; Yazar et al., 2003[34]). However, due to lack of well-matched case-control studies in large enough population of epileptic patients, there is not yet a consensus on association between epilepsy and latent *Toxoplasma* infection (Ngoungou et al., 2015[22]).

The seroprevalence of latent *Toxoplasma* infection among the general population in Iran was found 39.3 % in a recent meta-analysis study with the range 14-66 % between different regions of the country (Daryani et al., 2014[9]). Meanwhile, there are two studies on the rate of *Toxoplasma* infection in Iranian epileptic patients (Zibaei et al., 2011[35]; Allahdin et al., 2015[2]). Zibaei et al. (2011[35]) compared the rate of *Toxoplasma* infection in 85 epileptic patients with 85 healthy people in west of Iran. They reported higher rate of the infection in epileptic patients (14.1 %) compared to the healthy people (4.7 %). In contrast, results of a study from south of Iran (Allahdin et al., 2015[2]) indicated lower incidence of *Toxoplasma* infection in epileptic patients than healthy people (14.2 % versus 30.4 %). The low sample size, lack of aged-matched control group, and conflicting results in these two studies led us to conduct a study with larger sample size and age-matched control in the capital of Iran, Tehran, to evaluate the frequency and titer of serum anti-*T. gondii* IgG antibody in Iranian epileptic patients. 

The prevalence of an infectious disease in a population and the age of that population are among most useful data in epidemiological studies of the infectious diseases. The annual risk of infection (ARI) is a valuable index that specifies relationship between age and prevalence of disease in a population (Cauthen et al., 2002[8]). This index indicates the possibility that an individual without previous infection has of being infected during the course of a year. The other aim of our study was to estimate ARI for toxoplasmosis in our study population. 

## Materials and Methods

### Study population and subject selection 

The study was approved by the ethics committee of Pasteur Institute of Iran and conforms to Helsinki declaration of 1975 as revised in 2000 and 2008. The study population is composed of Iranian epileptic patients (IEP, n = 414), and non-epileptic patients (NEP) suffering from other neurologic disorders (n = 150) who referred to Loghman hospital (Tehran) throughout the country since February 2013 until January 2015. The age of participants was between 18 to 64 years. A control age-matched healthy people without any neurologic diseases (HP) group (n = 63) was also included in the study. The inclusion criteria were all adults of age 18-64 years, with any kind of epilepsy (IEP group), or other neurologic disorders except for epilepsy (NEP group), or healthy people without any neurologic diseases (HP group). The exclusion criteria were serum C reactive protein more than 10 mg/dL, immunodeficiency, concurrent consumption of immunosuppressive drugs, having a history of chemotherapy or immunotherapy during last 6 months, infectious disease, diabetes, malnutrition, and HIV.

The seizure type and etiology of epilepsy verified by neurologist Professor Kourosh Gharagozli according to the International League Against Epilepsy (ILAE) 2001 criteria (Shorvon, 2011[27]). 

All subjects participated in this study voluntarily. Written informed consent was obtained from all subjects following a complete description of the study. A 5 mL venous blood sample was taken for measuring the titer of anti-*Toxoplasma* antibody. Subject information and the data were identified by a code to ensure blind analysis.

### Serological antibody detection

Serum of the blood samples were isolated and kept at -20° C until use. The presence of anti-*Toxoplasma* IgG antibody in the sera was determined using commercial Euroimmun ELISA IgG kit (Euroimmun, Lübeck, Germany). The procedure was performed according to the manufacturer instruction. 

### Statistical analysis 

ARI is calculated by using the following equation; ARI = 1-(1-P)^1/M^, where P is the estimated seroprevalence of the infection, and M is mean age of intended population (Cauthen et al., 2002[8]). SPSS software version 22.0 (SPSS Inc. Chicago, Illinois) was used to analyze the data. The seroprevalence rate among the three groups was assessed with the two-tailed Pearson's Chi-*Square* test. The association of *T. gondii* infection and socio-demographic characteristics was assessed with the Pearson's Chi-*Square* test, and odds ratio (OR) and 95 % confidence interval (CI) were calculated. The difference between groups in quantitative variables was assessed using one way ANOVA. P < 0.05 was considered statistically significant.

## Results

**Figure 1:**
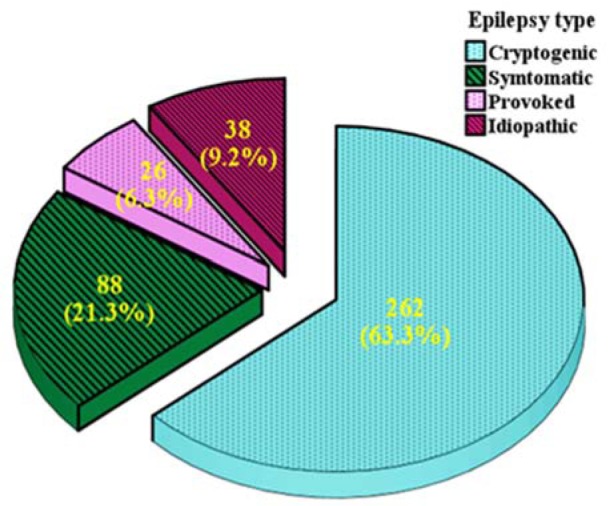
Distribution of epilepsy types in the epileptic population of the study

From 802 candidates, 627 people were entered in the study groups including IEP (n = 414), NEP (n = 150) and HP (n = 63). The seizure type and etiology of epilepsy in IEP group were shown in Figure 1(Fig. 1). The lowest and highest frequency of epilepsy belonged to provoked and cryptogenic types, respectively.

Demographic characteristics of the study population are shown in Table 1(Tab. 1). The study groups were matched by age as 18-30 years, 31-40 years, 41-50 years and 51 years and older. Distribution of male and female subjects in the population was significantly (P = 0.014) different in the three groups. The results showed that the marriage rate was low in the IEP group compared to the NEP and HP groups (P = 0.002). In terms of education, the population was categorized in three groups; school education, high school education and university education. The results showed that education level is significantly (P < 0.001) different between three groups. School education was higher in the IEP group while university education was higher in the NEP group.

As Tehran is the capital of Iran, many patients from other cities might refer to capital medical centers. Therefore, we assessed the number of capital residents in the study population. As it is seen in Table 1(Tab. 1), most of the people in the study groups were from Tehran, and there was no significant difference between groups in this regard.

Table 2(Tab. 2) shows the seroprevalence of latent *T. gondii* infection among the study groups. The seropositive rate in IEP group was not significantly different than that in the NEP and HP groups (P = 0.89). Though, the *Toxoplasma* seropositive rate was the least in idiopathic subgroup (23.7 %) and was the most in provoked subgroup (42.3 %), there was no significant difference between subgroups in this respect (P = 0.41). 

ELISA results showed that anti-*T. gondii* IgG titer were 55.7 ± 78, 52.4 ± 74 and 69.7 ± 92 IU/ml in the IEP, NEP and HP groups, respectively. The difference in the titer was not significant between groups (one-way ANOVA, P = 0.32).

Table 3(Tab. 3) demonstrates some socio-demographic characteristics of the study population and the seroprevalence of *T. gondii*. In our study, age was highly associated with the latent infection (P < 0.001). The IgG titer of *T. gondii* also significantly increased (P < 0.001, Pearson's r value of + 0.254*) *with age. The IgG titer for ≤ 30, 31-40, 41-50 and ≥ 51 year age groups were found as 44.6 ± 76, 54.4 ± 74, 74.8 ± 77 and 106.3 ± 84, respectively.

The total seroprevalence of *T. gondii* was found 0.3541 for study population. It means 35.4 % of whole population including IEP, NEP and HP groups was seropositive. 

The mean age of the population was 32.4 and ARI was obtained 0.0133. 

Our results showed no correlation between gender and seropositive rate of *T. gondii *infection (P = 0.61).

The married people had mean age of 37.4 with highly significant titer of *T. gondii* antibodies (P < 0.001) compared to single people (with mean age of 26.1).

Our results showed that latent *T. gondii* infection was negatively correlated (Pearson correlation of -0.112) with high education level (P = 0.006). 

Moreover, the results showed that consumption of undercooked meat and raw vegetables were highly correlated with *Toxoplasma* infection and considered to be a major risk factor (P < 0.001). However, outdoor eating habit (P = 0.17) or blood group (P = 0.64) did not increase the risk of *Toxoplasma* infection (Table 3(Tab. 3)). 

Our results showed that *Toxoplasma* seropositive people had high rate of visual anomalies (P = 0.04) compared to uninfected people. 

## Discussion

Results of the present study indicate that the seroprevalence of *Toxoplasma* in Iranian epileptic patients was not significantly different from non-epileptic patients and healthy control people. IgG seroprevalence was 35.3 %, 34.7 %, and 38.1 % in IEP, NEP, and HP, respectively. In term of epilepsy etiology, the seroprevalence of *Toxoplasma *infection was not significantly different among the epilepsy subgroups. Although obvious difference was seen in provoked and idiopathic subgroups, the small number of patients in these subgroups resulted in non-significant difference.

Numerous studies investigated the association between T. gondii infection and epilepsy (Akyol et al., 2007[1]; Allahdin et al., 2015[2]; El-Tantawy et al., 2013[11]; Eraky et al., 2016[12]; Ngugi et al., 2013[23]; Stommel et al., 2001[28]; Yazar et al., 2003[34]; Zibaei et al., 2011[35]). Some of these studies found significant association between epilepsy and *Toxoplasma* infection and some did not (Ngoungou et al., 2015[22]). Among these studies only one study found negative association between toxoplasmosis and epilepsy (Allahdin et al., 2015[2]). Most of these studies had small sample size except for Ngugi et al., (2013[23]) with 2262 subjects (971 epileptic patients). We also used a large sample size with 442 epileptic patients. However, we did not found any association between epilepsy and *Toxoplasma* infection. There are two studies, carried out in Iran on the rate of *T. gondii* infection in epileptic patients (Allahdin et al., 2015[2]; Zibaei et al., 2011[35]). In our study, the seroprevalence of *Toxoplasma* infection was found 35-38 % among epileptic, non-epileptic and control groups. This finding is consistent with the recent meta-analysis study which is reported a 39 % seroprevalence of *T. gondii* in Iranian general population (Daryani et al., 2014[9]). However, in the two previous studies on the rate of toxoplasmosis in Iranian epileptic patients, the seroprevalence rate was significantly different compared to the finding of the meta-analysis study reported by Daryani et al., (2014[9]). Zibaei et al. (2011[35]) reported 4.7 % seropositive rate in healthy people versus 14.1 % in epileptic patients. On the other hand, Allahadin et al. (2015[2]) found 30.4 % seropositive rate in healthy people versus 14.1 % in epileptic patients. Therefore, our results seem to be more reliable compared to studies performed by Zibaei et al. (2011[35]) and Allahadin et al. (2015[2]). 

There are several limitations in the study of Zibaei et al. (2011[35]) and Allahadin et al. (2015[2]). Zibaei et al. (2011[35]) used a small sample sizes (85 cases) and only cryptogenic epilepsy cases. Allahdin et al. (2015[2]) used higher sample size with 141 epileptic patients. However, they did not match the age of the study groups as the mean age of control cases was almost 10 years older than epileptic patients. It is well known that the rate of *Toxoplasma* infection is increased with age (Flegr et al., 2014[16]; Portela et al., 2004[25]). In our study, we matched the age of the subjects in the 3 groups of the study. In contrast to age, gender is not a risk factor for *Toxoplasma* infection worldwide (Flegr et al., 2014[16]) and also in Iran (Daryani et al., 2014[9]). Therefore, we did not match the gender of the groups in our study. The other strength of our study might be using two control groups i.e. non-epileptic patients that had neurologic disorders other than epilepsy, and healthy people with no disease. A positive correlation between *Toxoplasma* infection and some neurological disease such as psychosis has been reported (Fekadu et al., 2010[14]). Therefore, using a control group of the patients with neurologic disorders except for epilepsy, will give us a reliable comparison to differentiate the correlation of epilepsy (but not other neurological disorders) with *Toxoplasma* infection.

Several mechanisms, mostly obtained from animal studies, are postulated for potential association between toxoplasmosis and epilepsy. The most important mechanism is coming from high affinity of *T.* *gondii* to the excitable tissues including nervous system where the parasite forms tissue cysts. Recent evidence indicates that previous opinion regarding the silent status of the parasite in the brain is not true; rather *T. gondii* has a dynamic status inside the cysts (Watts et al., 2015[32]) thereby can change the function of the neurons by modulation of some neurotransmitters including GABA (Brooks et al., 2015[7]), dopamine (Babaie et al., 2017[3]), serotonin (Gatkowska et al., 2013[17]), noradrenaline (Gatkowska et al., 2013[17]), nitric oxide (Tonin et al., 2014[31]; Dincel and Atmaca 2015[10]), and glutamate (Kannan et al., 2016[21]). Moreover, serum level of pro-inflammatory cytokines also increases during *T. gondii* infection (Bottari et al., 2015[6]), which contribute to neuropathology of toxoplasmosis. Therefore, modulation of neurotransmitters and cytokine systems might be involved in the increased seizure susceptibility induced by *T. gondii*. The other main mechanism is the neuronal injury caused by the parasite. The injury is triggered either directly by rupture of the cysts, or indirectly by activation of the immune system and subsequent inflammation and tissue necrosis. Therefore, both immune response to the parasite invasion and altered neurotransmission by parasite itself can contribute to the development of epilepsy by toxoplasmosis.

There is some evidence that some antiepileptic drugs are able to inhibit *T. gondii* parasite. For instance, valproic acid, which is a broad-spectrum anticonvulsant drug efficiently inhibits *Toxoplasma* growth in vitro (Jones-Brando et al., 2003[19]). In addition carbamazepine, another anticonvulsant medication, had some effects on *T. gondii* growth (Jones-Brando et al., 2003[19]). Therefore, it is possible that the use of antiepileptic drugs might inhibit the activity of the parasite and affects seropositive rate of *T. gondii* in epileptic patients. Furthermore, many of epileptic patients start taking antiepileptic drugs from an early age that may protect them against *T. gondii* infection. 

We found that the prevalence of anti-toxoplasma antibodies significantly increased with age, which is in agreement with the results of other researchers (Bobic et al., 1998[5]; Daryani et al., 2014[9]; Ertug et al., 2005[13]; Jones et al., 2001[18]; Kamani et al., 2009[20]). Since diet changes with age, eating the raw or semi-cooked meat in adulthood increases the risk of exposure to the parasite. Thus, the prevalence of infection in adults is expected to be higher than that in children. On the other hand, consistent with other studies (Jones et al., 2001[18]), we found that low level of education is associated with the increased risk of infection with *T. gondii*. The knowledge on and the adherence to hygiene guidelines in the educated people are more than uneducated people. Most of educated people are well aware the modes of transmission of toxoplasmosis and therefore, are less prone to Toxoplasma infection. Hence, it is important that populations with high risk of infection are educated regarding the modes of transmission of toxoplasmosis and tips to prevent the spread of disease.

The annual risk of toxoplasmosis in the population of our study was estimated 1.33 %. This value reflects the probability that an individual without previous contact with *T. gondii* might acquire toxoplasmosis during the course of a year. ARI value is reported for some infectious diseases such as tuberculosis. However, we did not find any study reporting ARI for toxoplasmosis. Rather *the prevalence* is usually reported for toxoplasmosis. Therefore, we could not compare the ARI value obtained in our study with other similar studies. However, we found a 35-38 % seroprevalence for *Toxoplasma* infection in our study population, which is similar to the seroprevalence of *T. gondii* in Iranian general population reported in a recent meta-analysis study (Daryani et al., 2014[9]) .

In our study, Toxoplasma seropositive people had high rate of visual anomalies. This finding is in line with the finding of other similar studies (Saadatnia and Golkar, 2012[26]). It is shown that the most common finding in acquired, congenital and reactivated toxoplasmosis is ophthalmic infections such as retinochoroiditis, which leads to severe ocular abnormalities and even blindness (Weiss and Kim, 2007[33]). 

In conclusion, the rate of *T. gondii* infection in a population of Iranian epileptic patients was not higher than that of corresponding non-epileptic patients and healthy non-epileptic people. Prospective studies with large sample size in clinical subgroups of epileptic patients with well-matched control groups across the country are suggested to more clarify the impact of *T. gondii* on acquisition of epilepsy. 

## Conflict of interest

The authors declare that they have no conflict of interest.

## Figures and Tables

**Table 1:**
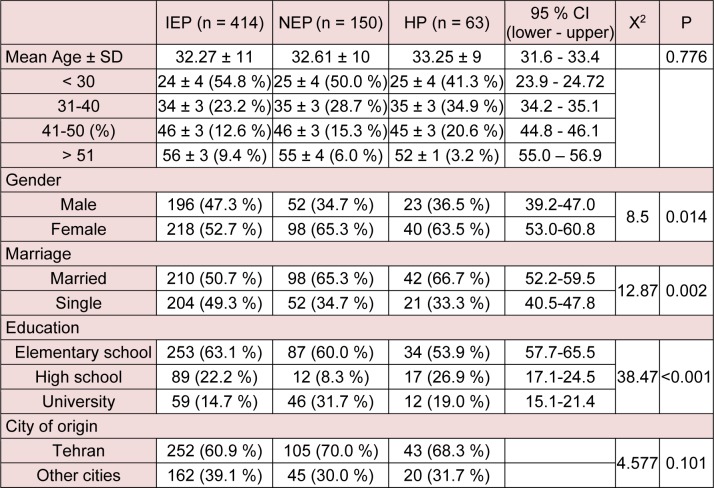
Demographic characteristics of the study population

**Table 2:**
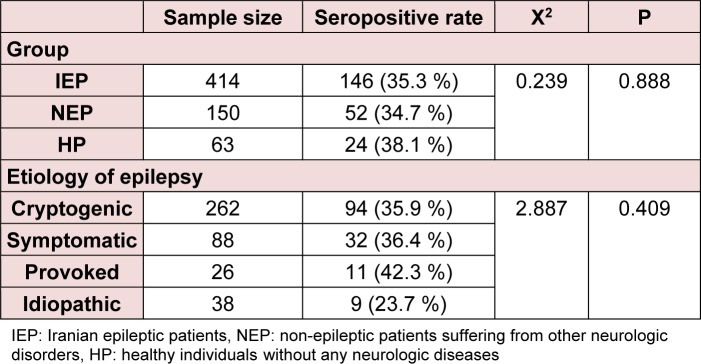
Seroprevalence of latent *T. gondii* infection in the epileptic population of the study

**Table 3:**
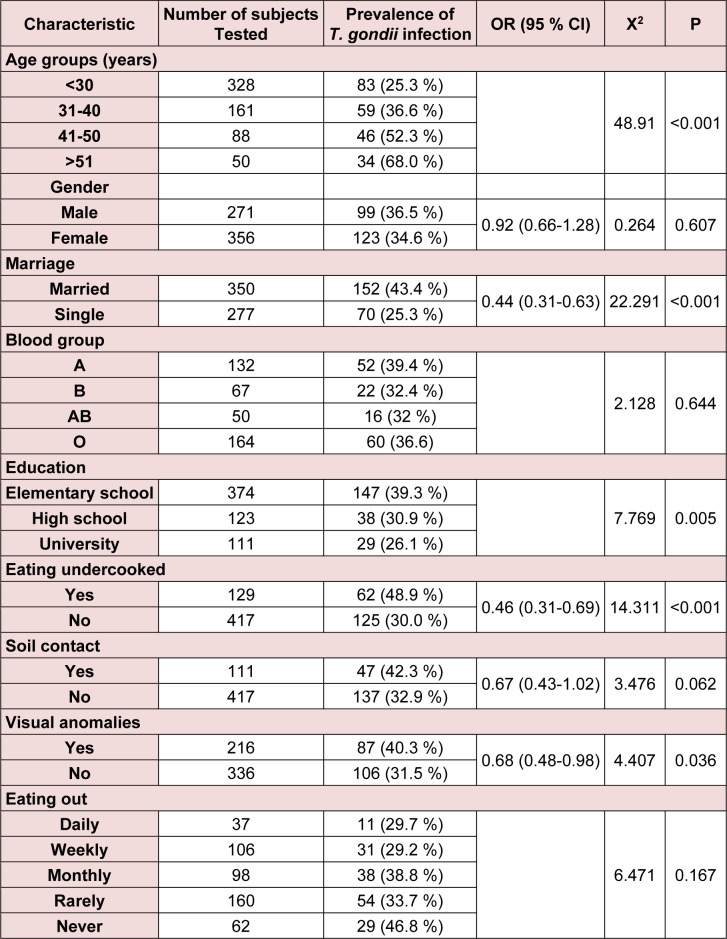
Socio-demographic characteristics and *T. gondii* seroprevalence in the study population
